# Liver stiffness in Fontan patients: the effect of respiration and food intake

**DOI:** 10.3389/fmed.2023.1192017

**Published:** 2023-09-07

**Authors:** Annabell Braun, Richard Mühlberg, Marcus Fischer, Nikolaus A. Haas, Zora Meyer

**Affiliations:** Department of Pediatric Cardiology and Pediatric Intensive Care, Hospital of the University of Munich, Ludwig Maximillian’s University Munich, Munich, Germany

**Keywords:** Fontan, liver, elastography, liver stiffness, respiration, food, ingestion, FibroScan

## Abstract

**Objectives:**

For several years, patients with single ventricle hearts have been palliated according to the Fontan principle. One well known long-term consequence in these patients is the Fontan-associated liver disease and fibrosis, which occurs due to the chronically increased Central Venous Pressure (CVP) after Fontan palliation. It carries an increased risk of liver cirrhosis and hepatocellular carcinoma over time. Liver elastography (LE) is a non-invasive, safe, and feasible ultrasound method to determine liver stiffness and the stage of liver fibrosis. Usually, this examination must be performed in a sober condition and strict inspiratory hold to optimize the results and may therefore be difficult to perform on children as a routine examination. However, the influence of food intake and respiration on these results in Fontan patients is unclear. To optimize the implementation for this examination especially in children, the effects of food intake and breathing maneuvers on liver stiffness in patients with Fontan circulation were investigated.

**Methods:**

For this prospective study, 25 Fontan patients (group 1) and 50 healthy volunteers (group 2) were examined. The two groups were additionally divided into two age categories (group 1a: 10–19  years; group 1b: 20–29  years; group 2a: 15–19  years; group 2b: 20–25  years). Liver stiffness was measured by liver elastography once before food intake (=T0, with 6 h of fasting). Subsequently the participants consumed a standardized chocolate drink (500  mL) with nutritional distribution corresponding to a standardized meal (600  kcal). Liver stiffness was then determined 15, 30, 45, 60, 90, 120, 150, and 180  min after ingestion. Each measurement of liver stiffness was performed during maximal inspiratory and expiratory holds. The study was reviewed and approved by the responsible ethics committee.

**Results:**

In group 2 there was a significant increase in liver stiffness after food intake at T15, T30, and T45 during inspiration measurements (T0 = 4.0 kPa vs. T15 = 4.9 kPa, difference = 22.5%; T0 = 4.0 kPa vs. T30 = 4.9 kPa difference = 22.5%; T0 = 4.0 kPa vs. T45 = 4.3 kPa difference = 7.5%), as well as during expiration at T15 and T30 (T0 = 4.5 kPa vs. T15 = 5.1 kPa, difference = 14.7%; T0 = 4.5 kPa vs. T30 = 4.9 kPa difference = 7.8%). Whereas in Fontan patients (group 1) liver stiffness did not differ significantly at any time between fasting (T0) and postprandial values. The respiratory maneuvers in the healthy subjects (group 2) differed significantly only before food intake (T0) (group 2: insp = 3.97 kPa vs. exp. = 4.48 kPa difference = 11.3%). In the Fontan group (group 1), there was no significant difference between the respiratory phases at any point. The different age categories showed no significant difference in liver stiffness.

**Conclusion:**

With these results we could demonstrate for the first time that in Fontan patients the time of food intake (i.e., fasting) has no clinical significance for the values obtained in liver elastography. We could also demonstrate that the breathing maneuvers during the examination had only minimal clinical impact on the results of liver elastography in patients with normal circulation and no effect in patients with Fontan-circulation. Consequently, liver elastography for Fontan patients is reliable independently of food intake and breathing maneuvers and can also be performed on younger patients, who are unable to follow breathing commands or longer fasting periods, without any impairment of the results. These results should encourage a routine use of LE in the follow-up of Fontan patients.

## 1. Introduction

Congenital heart defects represent 28% percent of all congenital malformations with an incidence of 11 of 1,000 newborns (1). 1.45% percent of these are univentricular in which an anatomical correction via surgery is not possible (1, 2). For palliative treatment, François M. F. Fontan developed the operation named after him in 1971, which aims to separate the pulmonary circulation from the systemic circulation. Thereby the mixture of oxygenated and deoxygenated blood is prevented and the univentricular heart relieved from volume overload (3, 4).

Due to the altered circulatory physiology in Fontan patients, caused by the cavopulmonary connection, the central venous pressure increases permanently, which leads to continuous hepatic congestion and thus to increased intrahepatic pressure. Histologically, the elevated central venous pressure leads to sinusoidal dilatation of the hepatocytes and increased filtration of the blood, resulting in edema in the hepatic sinusoids (5). These changes in the liver physiology and the increase in liver stiffness are well-known long-term consequences in patients with Fontan palliation and have been described in multiple studies (6–9).

In two independent studies, Munsterman et al. and Rathgeber et al. investigated the occurrence of Fontan-associated liver disease in children and young adults (7, 8). In 100% percent of the examined patients, they found fibrotic liver sinusoids (8, 9). For the diagnosis of this pathology, non-invasive methods such as specialized ultrasound methods exist today in addition to the classical biopsy. Among these methods, sonographic liver elastography (transient elastography, TE) has been established as a non-invasive and safely feasible method to assess the stiffness of the liver tissue. TE results were confirmed and validated using extensive data from cardiac catheters and liver biopsies (10).

Various factors are known to influence liver stiffness measurements, such as breathing, food intake or body posture. The influence of these factors has been demonstrated to affect TE results in both healthy and liver-diseased subjects. In general, patients with congenital heart disease were excluded from these study cohorts (11–13).

In two independent studies, Arena et al. and Berzigotti et al. examined the influence of a standardized meal in adult patients with chronic liver disease and demonstrated an increase in liver stiffness 30 min postprandially (14, 15).

The effect of the respiratory phases on liver stiffness has been evaluated by Ling et al. and Yun et al. (12, 16) They found that liver stiffness values differed between natural inspiratory (3.5 ± 1.1 kPa) and natural expiratory (4.3 ± 1.3 kPa) postures. Liver stiffness in end-expiration yielded significantly higher values (12, 16).

So far, no valid data exist on the above-mentioned effects on the liver stiffness in patients with Fontan circulation. It is conceivable that due to the different hemodynamics, the influence of food intake and breathing may have less effect on the values obtained in elastography. Therefore, the purpose of this study was to investigate and compare the effect of food intake and the effect of respiratory phases on liver stiffness in Fontan patients and a healthy comparison group.

## 2. Methods

### 2.1. Ethical statement

The study was reviewed and approved according to the guidelines of the Declaration of Helsinki by the responsible Ethics Committee of the Ludwig-Maximillian’s-University Munich (Munich, Germany) (Project no: 21-0165, date of approval: 05.07.2021) The data protection was observed in accordance with the guidelines of the Ethics Commission.

### 2.2. Study design and subject groups

**Figure 1:**
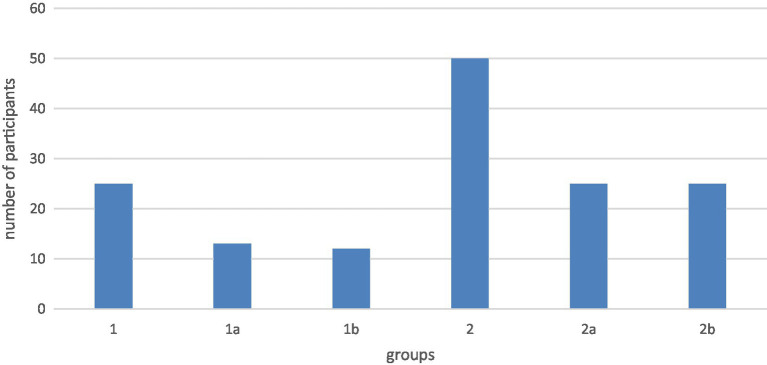
Age of the subgroups group 1a: 10–19 years; group 1b: 20–29 years; group 2a: 15–19 years; group 2b: 20–25 years.

This study is a clinical trial and diagnostic study. The group selection was not randomized and not blinded. The results were collected prospectively, and all study methods were standardized and performed by two investigators. Fontan Patients were recruited and invited to participate in the study from the registry of the Department of Paediatric Cardiology and Paediatric Intensive Care of the LMU. A total of 25 patients with Fontan circulation were recruited for the study (group 1). To show the effect of food intake and respiration at normal physiological conditions, the healthy comparison group consisted of 50 volunteers (group 2). To assess the effect of age on liver stiffness, both groups were divided into two subgroups of different ages (group 1a: 10–19 years; group 1b: 20–29 years; group 2a: 15–19 years; group 2b: 20–25 years) (Figure 1). All participants were informed in detail about the procedure and background of the study in advance and gave written informed consent.

### 2.3. Inclusion and exclusion criteria

Subjects aged between 10 and 30 years, with Fontan completion were recruited for group 1. There were no specific exclusion criteria for group 1. For group 2, healthy subjects aged between 10 and 30 years were recruited. Here for exclusion criteria were liver disease (e.g., liver fibrosis, liver cirrhosis, infection, and liver tumor), other vascular diseases, diseases of the cardiovascular system (e.g., cardiac arrhythmias, hypertension, and heart failure), metabolic diseases (e.g., hyperthyroidism and disorders of sugar metabolism), diseases of the gastrointestinal tract (e.g., gallbladder diseases, gallstones, and bile duct obstruction), diseases of the kidneys, diseases of the nervous system, mental diseases, regular drug use including nicotine and alcohol, obesity, subjects with regular medication use (exception: contraceptives), and pregnant women.

### 2.4. Sonography and elastography

After consent was obtained, vital signs and biometric data were recorded. In addition, blood flow in the hepatic vessels and the diameter of the inferior vena cava were determined in all subjects using a PW Doppler (Philips).

Subsequently, liver stiffness was measured once before food intake (=T0, fasting values) using elastography (FibroScan^®^ Echosens Paris, Philips). This was followed by ingestion of the liquid food, whose nutritional values correspond to a standardized meal (14, 15, 17). Liver stiffness was also measured 15, 30, 45, 60, 90, 120, 150, and 180 min after food intake. Liver stiffness was measured ten times at each time point in inspiratory and expiratory hold (2–4 min of measuring per time point). For the examination, the subjects were in supine position during the examinations with the transducer being placed in the right intercostal spaces.

### 2.5. Food intake

For the simulation of food intake, a 500 mL cocoa drink was used, based on comparable studies. The nutrient distribution is as follows: 600 kcal, 54% carbohydrates, 17% proteins and 29% fats. This distribution corresponds approximately to an average meal. This composition was based on previously published nutritional standards (14, 15, 17). For Fontan patients with bronchitis plastica, an alternative variant with low-fat milk and MTC oil was offered. The exact nutritional values can be found in Table 1.

**Table 1:** Nutrient distribution of the literature values and the beverage used.

		Calories (kcal)	Protein (g)	Carbohydrates (g)	Fat (g)
Reference value^a^		**600**	**25**	**80.8**	**19.6**
Chocolate drink	0,5 L milk^b^	330	16,5	25	18
	70 g cocoa^c^	260.4	8.68	51.94	1.4
	total	**590.4**	**25.18**	**76.94**	**19.4**
Alternative drink	0,5 L milk^d^	240	17	25.5	8
	70 g cocoa^c^	260.4	8.68	51.94	1.4
	MCT oil	89.41	0	0	10.1
	total	**589.81**	**25.68**	**77.44**	**19.51**

a 400ml Ensure^®^ Plus.

b cow milk with 3.5% fat.

c Ovomaltine^®^.

d cow milk with 1.5% fat.The bold values represent the total nutritional values of the liquid foods compared.

### 2.6. Study parameters

- General medical history (previous illness/diagnosis, age).
- Biometric data (height, weight, BMI).
- Blood pressure measurement, heart rate, oxygen saturation.
- Liver stiffness before and after food intake.
- Blood flow in the vena portae, venae hepaticae, truncus coeliacus (presented as Supplementary data in Supplementary material).
- Diameter of inferior vena cava (presented as Supplementary data in Supplementary material).

### 2.7. Statistical processing of data

Statistical analysis was performed using the Statistical Package for Social Sciences software (IBM SPSS version 26.0.0.1). In the context of descriptive statistics, mean and standard deviation were used to describe quantitative characteristics. Where values were not based on a normal distribution, median and interquartile range were used. In a T-test for unrelated target variables and a repeated measures ANOVA, values of inspiration and expiration were compared. For comparison of fasting and postprandial liver stiffness values a T-test for linked target values was applied. Statistically significance was set to a two-sided value of *p* <0.05. To all results the Bonferroni correction was applied.

## 3. Results

### 3.1. Patient characteristics

A total of 25 Fontan patients and 50 healthy subjects were included in the data analysis. The distribution of patient characteristics and vital signs are shown in Table 2. There were significant differences in the categories weight (*p* = 0.008), heart rate (*p* = 0.046) and oxygen saturation (*p* < 0.001). Liver elastography was successfully performed in each participant. Liver stiffness is expressed in units of kPa.

**Table 2:** Descriptive statistics of main groups 1 vs. 2.

Variable	Group 1 (*n* = 25)	Group 2 (*n* = 50)	*p* value
Age (years)^a^	18 (8)	20 (6)	0.142
Height (cm)	168 (14.5)	173 (10.2)	0.084
Weight (kg)	56 (16.5)	65 (12.7)	0.008*
BMI (kg/m^2^)	20 (2.8)	22 (4.1)	0.068
Systolic blood pressure (mmHg)	120 (12.6)	116 (7.3)	0.155
Diastolic blood pressure (mmHg)	72 (10.5)	74 (6.3)	0.361
Heart rate (/min)	89 (14.2)	73 (11.9)	0.046*
Oxygen saturation (%)	92 (4.2)	98 (0.9)	<0.001*

BMI Body mass index, ^a^Age is described by median and IQR due to bimodal distribution of values, *Statistically significant.

**Figure 2:**
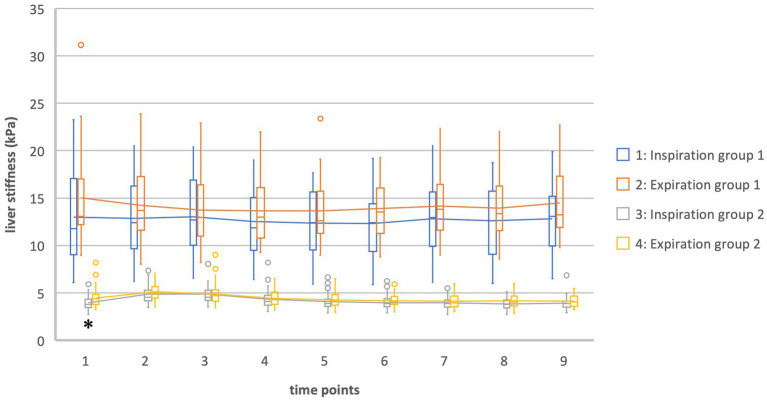
Liver stiffness of groups 1 and 2 in inspiration vs. expiration *Statistically significant.

In general, values for liver stiffness were significantly different in group 1 and 2. At T0 in inspiration there was a difference of 9.01 kPa [T (24.7) = 9.36, *p* = <0.002] while at T0 in expiration there was a difference of 10.54 kPa [T (24.9) = 10.69, *p* = <0.002] (Table 3; Figure 2). In group 1 our data showed a higher standard deviation than in group 2.

**Table 3:** Liver stiffness group 1 vs. group 2 at T0.

	Group 1 mean (SD)	Group 2 mean (SD)	Relative change (%)	*p* value
Inspiration	12.98 (4.8)	3.97 (0.8)	69.45	< 0.002*
Expiration	15.01 (4.9)	4.48 (0.9)	70.2	< 0.002*

SD standard deviation, *Statistically significant.

### 3.2. Food intake

#### 3.2.1. Group 2

**Figure 3:**
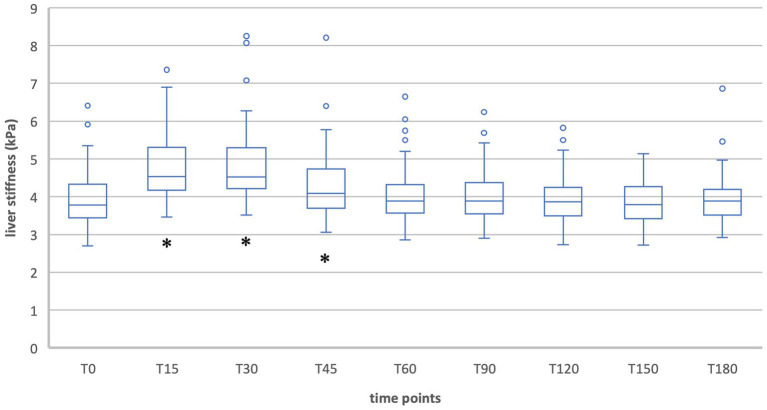
Liver stiffness during fasting vs. after food intake in group 2 during inspiration. *Statistically significant.

**Figure 4:**
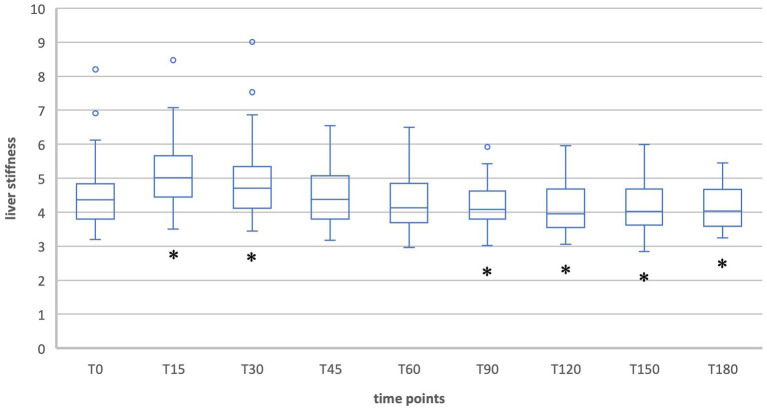
Liver stiffness during fasting vs. after food intake in group 2 during expiration. *Statistically significant.

During inspiration in group 2 the measurements showed a significant increase in liver stiffness 15, 30 and 45 min after food intake [T15: T(49) = −11.19, *p* < 0.001; T30: T(49) = −8.14, *p* < 0.001; T45: T(49) = −3.16, *p* = 0.003]. At T30 liver stiffness was at its maximum (T30 = 4.9 kPa). From 60 min after food intake, liver stiffness was no longer above baseline and no more significant differences occurred (Table 4, Figures 3, 4).

**Table 4:** Liver stiffness during fasting vs. after food intake in group 2.

	Time points	Mean/kPa (SD)	Postprandial change/kPa (SD)	Relative change (%)	*p* value
Inspiration	T0	4,0 (0,81)			
	T15	4,9 (0,95)	−0,9 (0,56)	22.5	< 0.001*
	T30	4,9 (1,04)	−0,9 (0,78)	22.5	< 0.001*
	T45	4,3 (0,91)	−0,3 (0,77)	7.5	0.024*
	T60	4,0 (0,76)	−0,1 (0,66)	2.5	> 0.999
	T90	4,0 (0,65)	0,0 (0,62)	0.0	> 0.999
	T120	4,0 (0,64)	0,0 (0,55)	0.0	> 0.999
	T150	3,9 (0,61)	0,1 (0,60)	2.5	> 0.999
	T180	3,9 (0,67)	0,0 (0,57)	0.0	> 0.999
Expiration	T0	4,5 (0,94)			
	T15	5,1 (0,96)	−0,66 (0,55)	14.7	< 0.001*
	T30	4,9 (1,04)	−0,38 (0,74)	7.8	0.005*
	T45	4,4 (0,74)	0,04 (0,72)	0.9	> 0.999
	T60	4,3 (0,83)	0,21 (0,64)	4.7	0.176
	T90	4,2 (0,63)	0,29 (0,69)	6.5	0.04*
	T120	4,1 (0,68)	0,35 (0,64)	7.8	0.002*
	T150	4,2 (0,66)	0,31 (0,54)	6.9	0.002*
	T180	4,1 (0,58)	0,36 (0,63)	8.0	0.001*

SD Standard deviation, *Statistically significant.

When liver stiffness was measured in expiration hold, a significant increase was shown 15 and 30 min postprandial [T15: T(49) = −8.61, *p* < 0.001; T30: T(49) = −3.68, *p* < 0.001]. A significant decrease compared to the fasting value was shown at T90, T120, T150, and T180 [T90: T(49) = 2.93, *p* = 0.005; T120: T(49) = 3,90, *p* < 0.001; T150: T(49) = 4.01, *p* < 0.001; T180: T(49) = 4.10, *p* < 0.001]. At the timepoints T45 and T60 the liver stiffness did not differ significantly compared to T0.

#### 3.2.2. Group 1

**Figure 5:**
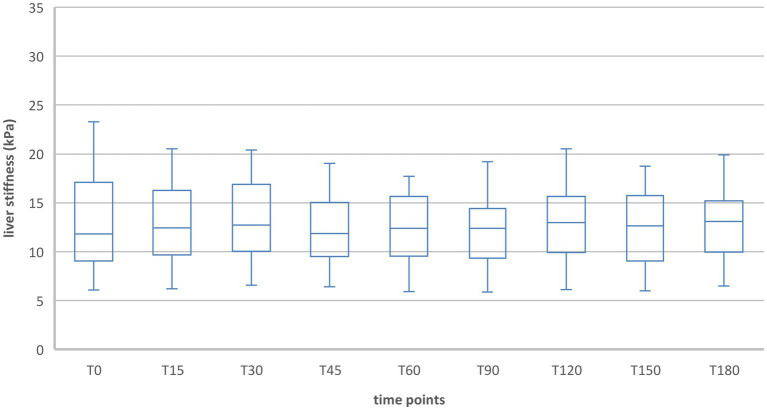
Liver stiffness during fasting vs. after food intake in group 1 during inspiration.

**Figure 6:**
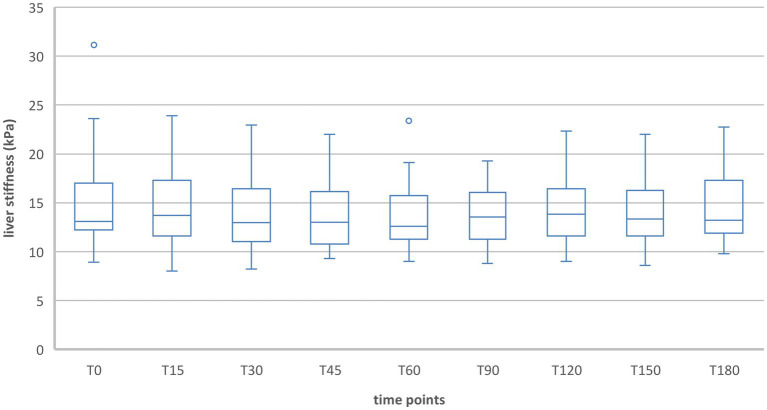
Liver stiffness during fasting vs. after food intake in group 1 during expiration.

When comparing liver stiffness during fasting and after food intake in the Fontan group (group 1), the values did not differ significantly at any time, neither in inspiration nor in expiration (Table 5; Figures 5, 6). No increase was shown at any time point compared to the fasting value (T0). The maximum change in liver stiffness occurred in inspiration at 90 min (0.6 kPA = 4.6%) and in expiration after 60 min (1.37 kPa = 9.1%).

**Table 5:** Liver stiffness during fasting vs. after food intake in group 1.

	Time points	Mean/kPa (SD)	Postprandial change/kPa (SD)	Relative change (%)	*p* value
Inspiration	T0	13,0 (4,78)			
	T15	12,9 (3,99)	0,1 (2,22)	0,8	> 0.999
	T30	13,0 (4,19)	0,0 (2,15)	0,0	> 0.999
	T45	12,5 (3,62)	0,4 (3,00)	3,1	> 0.999
	T60	12,4 (3,52)	0,6 (2,75)	4,6	> 0.999
	T90	12,3 (3,68)	0,6 (3,18)	4,6	> 0.999
	T120	12,8 (3,80)	0,2 (3,35)	1,5	> 0.999
	T150	12,6 (3.86)	0,3 (2,80)	2,3	> 0.999
	T180	12,8 (3,41)	0,2 (3,29)	1,5	> 0.999
Expiration	T0	15,0 (4,88)			
	T15	14,2 (3,97)	0,78 (2,63)	5,2	> 0.999
	T30	13,7 (3,56)	1,28 (2,89)	8,5	0.288
	T45	13,6 (3,37)	1,36 (3,31)	9,0	0.408
	T60	13,6 (3,36)	1,37 (3,21)	9,1	0.344
	T90	13,9 (3,35)	1,12 (3,06)	7,5	0.632
	T120	14,1 (3,19)	0.87 (3,53)	5,8	> 0.999
	T150	13,9 (3,26)	1,08 (3,67)	7,2	0.984
	T180	14,5 (3,37)	0,50 (3,72)	3,3	> 0.999

SD standard deviation.

### 3.3. Respiration

In group 2 there was a significant difference between the values measured in inspiration and expiration at T0 (fasting value). Liver stiffness at inspiration was measured at 3.97 kPa, while at expiration it was measured at 4.48 kPa [difference 0.51 kPa = 11.3%, T (98) = −2.9, *p* = 0.045]. For the other times of measurement, the T-Test showed no significance between inspiration and expiration (Tables 6, 7; Figure 2).

**Table 6:** Liver stiffness inspiration vs. expiration for group 1 and 2 at T0.

	Inspiration (kPa) mean (SD)	Expiration (kPa) mean (SD)	Relative change (%)	*p* value
Group 1	12.98 (4.8)	15.01 (4.9)	13.5	0.145
Group 2	3.97 (0.8)	4.48 (0.9)	11.3	0.01*

*Statistically significant.

**Table 7:** Liver stiffness in inspiration vs. expiration group 2.

	Inspiration mean (SD)	Expiration mena (SD)	*p* value
T0	3.97 (0.8)	4.47 (0.9)	0.045*
T15	4.86 (1.0)	5.14 (1.0)	0.93
T30	4.87 (0.9)	4.86 (1.0)	0.968
T45	4.32 (0.9)	4.43 (0.7)	0.968
T60	4.07 (0.8)	4.26 (0.8)	0.93
T90	3.97 (0.7)	4.19 (0.6)	0.609
T120	3.96 (0.6)	4.12 (0.7)	0.93
T150	3.85 (0.6)	4.17 (0.7)	0.12
T180	3.94 (0.7)	4.11 (0.6)	0.93

*Statistically significant.

Overtime, the difference between inspiration and expiration was analyzed in a repeated measures ANOVA. In group 2 there was a significant change of the difference between inspiration and expiration between T0 and T30 (*p* = 0.003) and T0 and T120 (*p* = 0.045) [*F* (5.3, 259.32) = 3.55, *p* = 0.003].

Meanwhile, in group 1 there was no significant difference between the values for liver stiffness in inspiration and expiration at any time point of the examination [e.g., T0 insp = 12.98 kPa; T0 exp. = 15.01 kPa; difference 2.03 kPa = 13.5%, T (−1.5) = 48, *p* = 0.145] (Tables 6, 8; Figure 2).

**Table 8:** Liver stiffness in inspiration vs. expiration group 1.

	Inspiration mean (SD)	Expiration mean (SD)	*p* value
T0	12.98 (4.8)	15.01 (4.9)	>0.999
T15	12.86 (4.0)	14.23 (4.0)	>0.999
T30	13.01 (4.2)	13.72 (3.6)	>0.999
T45	12.54 (3.6)	13.65 (3.4)	>0.999
T60	12.38 (3.5)	13.63 (3.4)	>0.999
T90	12.34 (3.7)	13.89 (3.4)	>0.999
T120	12.80 (3.8)	14.14 (3.2)	>0.999
T150	12.63 (3.9)	13.93 (3.3)	>0.999
T180	12.81 (3.4)	14.50 (3.4)	0.765

The repeated measures ANOVA in group 1 also showed no significant changes in the difference between inspiration and expiration overtime.

### 3.4. Age

**Figure 7:**
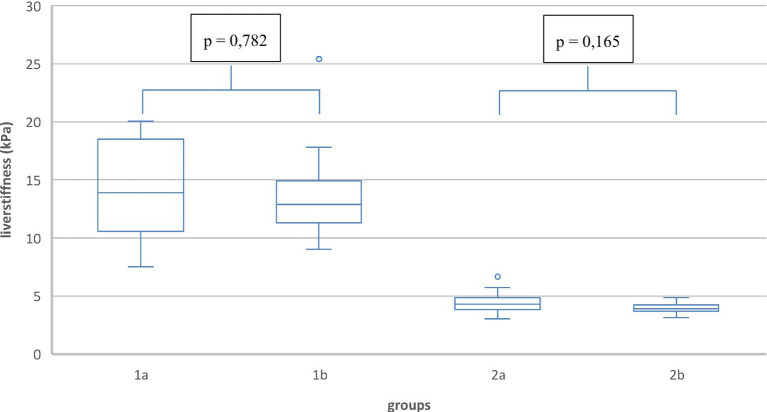
Liver stiffness (T0) in the subgroups of different age.

The main groups were divided into two subgroups of different ages. Thus, the effect of age on liver stiffness (T0) could be determined in Fontan patients and in healthy subjects. The comparison of liver stiffnesses during fasting in group 1a (14.2 kPa) and in group 1b (13.7 kPa) showed no statistically significant difference (Figure 7). The liver stiffnesses of group 2a (4.4 kPa) and group 2b (4.1 kPa) was also not significantly different. In addition, the regression analyses did not reveal a statistically significant correlation between liver stiffness and age in any group (group 1: R2 = 0.011; group 2: R2 = 0.057).

## 4. Discussion

### 4.1. FALD and liver elastography

Due to the lack of a subpulmonary ventricle, Fontan patients routinely present with increased central venous pressure and blood backing up into the liver. These factors can lead to complications such as Fontan-associated liver disease (FALD), bronchitis plastica, protein-losing enteropathy, and even failing Fontan during its course (18).

Beside the gold standard, i.e. liver biopsy, liver ultrasonography is used in the follow-up of Fontan patients as a diagnostic procedure for liver fibrosis and cirrhosis. It is a non-invasive and safe procedure that measures liver stiffness sonographically (10). In patients with a healthy heart and circulation, this procedure has been adequately evaluated for accuracy, confounding variables, and utility (19). The results of liver stiffness and biopsy were compared in 100 subjects by Jain. et al. Here, liver elastography was found to have a sensitivity of 90.3% and a specificity of 89.5% in the presence of significant fibrosis (*F* > 2) (20). Also, Castera et al. and and Foucher et al. defined cutoff values for the stages of fibrosis based on the METAVIR Fibrose-Score. The described values were for F0/F1 = 2.5–7.2 kPa, F2 = 7.2–12.5 kPa, F3 = 12.5–17.6 kPa, F4 = > 17.6 kPa (21, 22). Among the confounding variables identified are food intake, respiration, and age. For example, the research by Berzigotti et al. showed a postprandial increase in liver stiffness from 40.7 kPa to 51.2 kPa 30 min after food intake (15). For this reason, it is currently recommended that liver elastography is performed in a fasting state (23, 24). In addition, Yun et al. found a significant difference in liver stiffness between expiratory and inspiratory phases (exp: 8.7 kPa vs. insp: 7.9 kPa) in their study of 123 subjects (16). The effect of age on liver stiffness is weighted differently in current studies and remains incompletely elucidated (25, 26). In Fontan patients, neither the influence of age nor the influence of respiration and food intake on liver stiffness has been validated so far.

### 4.2. Food intake

To address these questions, our study examined 25 patients after Fontan completion by liver elastography in the fasting state and after ingestion of a standardized meal and in the inspiratory and expiratory positions. For comparison, a group of 50 healthy subjects was examined under the same conditions.

The healthy subjects showed a typical postprandial increase in liver elastography values that was significant for the first 45 min during inspiration measurements and for the first 30 min during expiration measurements. The maximum was reached after 30 min (T30 = 4.9 kPa) in inspiration and after 15 min (T15 = 5.1 kPa) in expiration position. This corresponds to relative changes of 22.5% and 14.7% and indicates a clinically relevant difference. In contrast, the decreases in liver stiffness during expiration measurements at the time points T90 (6.5%), T120 (7.8%), T150 (6.9%), and T180 (8.0%) were clinically negligible. The increase in liver stiffness after meal ingestion has been described in other studies and can be explained by increased postprandial blood flow to the liver (14, 15, 23). Thus, also Alvarez et al. showed an increase from 7.8 kPa to 10.3 kPa 30 min postprandially in adult patients (23).

In contrast, the group of Fontan patients did not show a significant change in liver stiffness at any time point after food intake, neither in inspiration nor in expiration measurements. These results can be explained by the fact that in Fontan patients, due to chronic blood stasis in the liver, postprandial hyperemia cannot contribute to increased liver stiffness. The study presents the first scientific documentation of the lack of increased liver stiffness following food intake in Fontan patients.

### 4.3. Respiration

For each of the nine measurement timepoints, liver stiffness was evaluated in inspiration and expiration. Physiologically, during inspiration the thoracic and intrapleural pressure lowers by expansion of the thorax through the breathing muscles, which creates an increase of venous return to the right heart (27). By Yun et al. it was reported that this increase in venous return leads to a significantly higher flow velocity in the hepatic veins than during expiration, where thoracic pressure rises leading to a decrease of blood flow to the heart and thereby leading to an elevation in intrahepatic blood volume which ultimately results in an increase of liver stiffness, that can be measured (12, 16).

As seen in our study, values for liver stiffness in expiration were generally higher than values measured in inspiration, which is consistent with results of other studies (12, 16). Values measured in group 2 showed a significant difference between inspiration and expiration at T0 (T0 insp = 3.97 kPa, T0 exp. = 4.48 kPa, difference 0.51 kPa). Except for the fasting value, no other measurement time point showed any significant difference. This could be influenced by the additional effect of meal ingestion on liver stiffness.

The Fontan patients in group 1 showed no significant difference between inspiration and expiration at any point during the study. Since the Fontan’s physiology leads to a chronically increased Central Venous Pressure it is possible that the physiological pressure gradients during inspiration and expiration do not affect the liver stiffness in Fontan patients. However, the lack of a significant difference between the values for inspiration and expiration in group 1 could also be due to the higher standard deviation. For a more detailed evaluation of this, further studies with a bigger sample size need to be performed.

These results could mean that liver elastography does not have to be performed in a specific phase of respiration in Fontan patients and is therefore also applicable to smaller children and patients who cannot follow breathing commands, with the results still being reliable.

### 4.4. Age

No significant difference in liver stiffness was shown between the subgroups. In our patient collective, there was no correlation between age and liver stiffness. This correlation is evaluated differently on healthy subjects in the current data (25, 26, 28). Tokuhara et al. studied preschoolers, elementary school students, and adolescents and described a statistically significant correlation between age and liver stiffness. The values increased from 3.4 kPa to 3.8 kPa and 4.1 kPa (25). In contrast, Sirli et al. described no age differences with respect to liver elastography results (28). However, no Fontan patients were studied here. In subjects with Fontan circulation, one suspects an increasing liver stiffness during life due to the chronic progressive hemodynamic influences. Here for further studies with a larger patient population of Fontan patients are necessary.

## 5. Conclusion

In contrast to the healthy comparison cohort, no variations in liver stiffness measured by ultrasound elastography were found in the Fontan patients under the influence of food intake and respiration. To the best of our knowledge, this is the first time that the clinical irrelevance of food intake and respiration in liver elastography of Fontan patients has been demonstrated. Especially for children this means a considerable relief for the future performance of liver elastography on Fontan patients, because fasting and respiratory maneuvers are no longer mandatory. To further optimize the follow-up of Fontan patients, additional studies on the performance and influencing factors of liver elastography are necessary. Furthermore, multicenter studies are important to establish cut-off values of liver stiffness depending on age and Fontan pathology.

### 5.1. Limitations

There are several limitations to this study. A total of 25 Fontan patients were recruited from the registry of the Department of Pediatric Cardiology and Pediatric Intensive Care of the LMU. This number can be considered relatively small. Therefore, no statements could be made about the differences in the subtypes of the univentricular heart disease. The sample size calculation was based in values from the available literature. Since there are no studies on changes in liver stiffness during food intake and respiration in Fontan patients, the standard deviation was chosen smaller than what was later found in our data. Because of the small prevalence of Fontan patients, multicenter studies are needed for this. In addition, the amount of the drink was not adapted to the characteristics of the subjects. Younger patients with a lower BMI may respond differently to food intake than older patients with a higher BMI. Furthermore, the breathing maneuvers were performed by the subjects themselves and could therefore not be accurately compared.

## Data availability statement

The original contributions presented in the study are included in the article/Supplementary materials, further inquiries can be directed to the corresponding author.

## Ethics statement

The studies involving human participants were reviewed and approved by Ethikkommission bei der LMU München (Project Number: 21-0165). Written informed consent to participate in this study was provided by the participants’ legal guardian/next of kin.

## Author contributions

NH and MF designed the study and conceptualized the manuscript. ZM accompanied the data collection and took care of the approval of the ethics application. AB and RM conducted the research, analyzed the data, and wrote the manuscript. NH, MF, and ZM reviewed the manuscript. All authors contributed to the article and approved the submitted version.

## Conflict of interest

The authors declare that the research was conducted in the absence of any commercial or financial relationships that could be construed as a potential conflict of interest.

## Publisher’s note

All claims expressed in this article are solely those of the authors and do not necessarily represent those of their affiliated organizations, or those of the publisher, the editors and the reviewers. Any product that may be evaluated in this article, or claim that may be made by its manufacturer, is not guaranteed or endorsed by the publisher.

## References

[1] van der Linde D Konings EE Slager MA Witsenburg M Helbing WA Takkenberg JJ. Birth prevalence of congenital heart disease worldwide: a systematic review and meta-analysis. J Am Coll Cardiol. (2011) 58:2241–7. doi: 10.1016/j.jacc.2011.08.025 22078432

[2] Liu Y Chen S Zühlke L Black GC Choy MK Li N. Global birth prevalence of congenital heart defects 1970-2017: updated systematic review and meta-analysis of 260 studies. Int J Epidemiol. (2019) 48:455–63. doi: 10.1093/ije/dyz009, PMID: 30783674 PMC6469300

[3] Jones MB. The Fontan Procedure for Single-Ventricle Physiology. Crit Care Nurse. (2018) 38:e1–e10. doi: 10.4037/ccn2018994 29437083

[4] Kverneland LS Kramer P Ovroutski S. Five decades of the Fontan operation: a systematic review of international reports on outcomes after univentricular palliation. Congenit Heart Dis. (2018) 13:181–93. doi: 10.1111/chd.12570, PMID: 29372588

[5] Téllez L Rodríguez-Santiago E Albillos A. Fontan-associated liver disease: a review. Ann Hepatol. (2018) 17:192–204. doi: 10.5604/01.3001.0010.8634 29469053

[6] Gordon-Walker TT Bove K Veldtman G. Fontan-associated liver disease: a review. J Cardiol. (2019) 74:223–32. doi: 10.1016/j.jjcc.2019.02.016 30928109

[7] Munsterman ID Duijnhouwer AL Kendall TJ Bronkhorst CM Ronot M van Wettere M. The clinical spectrum ofFontan-associated liver disease: results from a prospective multimodality screening cohort. Eur Heart J. (2019) 40:1057–68. doi: 10.1093/eurheartj/ehy620, PMID: 30346512

[8] Rathgeber SL Guttman OR Lee AF Voss C Hemphill NM Schreiber RA. Fontan-associated liver disease: Spectrum of disease in children and adolescents. J Am Heart Assoc. (2020) 9:e012529. doi: 10.1161/JAHA.119.012529 31902322 PMC6988152

[9] Andersen SB Ewertsen C Carlsen JF Henriksen BM Nielsen MB. Ultrasound Elastography is useful for evaluation of liver fibrosis in children-a systematic review. J Pediatr Gastroenterol Nutr. (2016) 63:389–99. doi: 10.1097/MPG.0000000000001171, PMID: 26925609

[10] Chen B Schreiber RA Human DG Potts JE Guttman OR. Assessment of Liver Stiffness in Pediatric Fontan Patients Using Transient Elastography. Can J Gastroenterol Hepatol. (2016) 2016:7125193–7. doi: 10.1155/2016/7125193, PMID: 27656638 PMC5021462

[11] Goertz RS Egger C Neurath MF Strobel D. Impact of food intake, ultrasound transducer, breathing maneuvers and body position on acoustic radiation force impulse (ARFI) elastometry of the liver. Ultraschall Med. (2012) 33:380–5. doi: 10.1055/s-0032-1312816, PMID: 22723037

[12] Ling W Lu Q Quan J Ma L Luo Y. Assessment of impact factors on shear wave based liver stiffness measurement. Eur J Radiol. (2013) 82:335–41. doi: 10.1016/j.ejrad.2012.10.004, PMID: 23116805

[13] Silva M Costa Moreira P Peixoto A Santos AL Lopes S Gonçalves R. Effect of Meal Ingestion on Liver Stiffness and Controlled Attenuation Parameter. GE Port J Gastroenterol. (2019) 26:99–104. doi: 10.1159/000488505, PMID: 30976614 PMC6454378

[14] Arena U Lupsor Platon M Stasi C Moscarella S Assarat A Bedogni G. Liver stiffness is influenced by a standardized meal in patients with chronic hepatitis C virus at different stages of fibrotic evolution. Hepatology. (2013) 58:65–72. doi: 10.1002/hep.26343, PMID: 23447459

[15] Berzigotti A De Gottardi A Vukotic R Siramolpiwat S Abraldes JG García-Pagan JC. Effect of meal ingestion on liver stiffness in patients with cirrhosis and portal hypertension. PLoS One. (2013) 8:e58742. doi: 10.1371/journal.pone.0058742, PMID: 23520531 PMC3592829

[16] Yun MH Seo YS Kang HS Lee KG Kim JH An H. The effect of the respiratory cycle on liver stiffness values as measured by transient elastography. J Viral Hepat. (2011) 18:631–6. doi: 10.1111/j.1365-2893.2010.01376.x, PMID: 21029256

[17] Ratchatasettakul K Rattanasiri S Promson K Sringam P Sobhonslidsuk A. The inverse effect of meal intake on controlled attenuation parameter and liver stiffness as assessed by transient elastography. BMC Gastroenterol. (2017) 17:50. doi: 10.1186/s12876-017-0609-6, PMID: 28407734 PMC5390386

[18] Schumacher KR Stringer KA Donohue JE Yu S Shaver A Caruthers RL. Fontan-associated protein-losing enteropathy and plastic bronchitis. J Pediatr. (2015) 166:970–7. doi: 10.1016/j.jpeds.2014.12.068, PMID: 25661406 PMC4564862

[19] Ferraioli G Wong VWS Castera L Berzigotti A Sporea I Dietrich CF. Liver ultrasound Elastography: An update to the world Federation for Ultrasound in medicine and biology guidelines and recommendations. Ultrasound Med Biol. (2018) 44:2419–40. doi: 10.1016/j.ultrasmedbio.2018.07.008, PMID: 30209008

[20] Jain V Poddar U Negi TS Saraswat VA Krishnani N Yachha SK. Utility and accuracy of transient elastography in determining liver fibrosis: a case-control study. Eur J Pediatr. (2020) 179:671–7. doi: 10.1007/s00431-019-03561-y, PMID: 31960149

[21] Foucher J Chanteloup E Vergniol J Castéra L Le Bail B Adhoute X. Diagnosis of cirrhosis by transient elastography (FibroScan): a prospective study. Gut. (2006) 55:403–8. doi: 10.1136/gut.2005.069153, PMID: 16020491 PMC1856085

[22] Castera L Forns X Alberti A. Non-invasive evaluation of liver fibrosis using transient elastography. J Hepatol. (2008) 48:835–47. doi: 10.1016/j.jhep.2008.02.008 18334275

[23] Alvarez D Orozco F Mella JM Anders M Antinucci F Mastai R. Meal ingestion markedly increases liver stiffness suggesting the need for liver stiffness determination in fasting conditions. Gastroenterol Hepatol. (2015) 38:431–5. doi: 10.1016/j.gastrohep.2015.01.009, PMID: 25769876

[24] Hui Z Jinlin H Jidong J Lai W Hong R Guiqiang W. Expert panel on liver stiffness measurement. Clinical application of transient Elastography in the diagnosis of liver fibrosis: an expert panel review and opinion. J Clin Transl Hepatol. (2014) 2:110–6. doi: 10.14218/JCTH.2014.00008 26357622 PMC4521263

[25] Tokuhara D Cho Y Shintaku H. Transient Elastography-based liver stiffness age-dependently increases in children. PLoS One. (2016) 11:e0166683. doi: 10.1371/journal.pone.0166683, PMID: 27861607 PMC5115769

[26] Kim SU Choi GH Han WK Kim BK Park JY Kim DY. What are 'true normal' liver stiffness values using FibroScan?: a prospective study in healthy living liver and kidney donors in South Korea. Liver Int. (2010) 30:268–74. doi: 10.1111/j.1478-3231.2009.02172.x, PMID: 19929903

[27] Laohachai K Ayer J. Impairments in pulmonary function in Fontan patients: their causes and consequences. Front Pediatr. (2022) 10:825841. doi: 10.3389/fped.2022.825841, PMID: 35498782 PMC9051243

[28] Sirli R Sporea I Tudora A Deleanu A Popescu A. Transient elastographic evaluation of subjects without known hepatic pathology: does age change the liver stiffness? J Gastrointestin Liver Dis. (2009) 18:57–60. Available at: https://www.jgld.ro/jgld/index.php/jgld/article/view/2009.1.9 (Accessed July 4, 2023). PMID: 19337635

