# Supplementary material for: Liver stiffness in Fontan patients: the effect of respiration and food intake
Source: Front Med (Lausanne). 2023 Sep 7;10:1192017. doi: 10.3389/fmed.2023.1192017 (PMC10512863; doi:10.3389/fmed.2023.1192017)
Supplement: Supplementary file 1 [file Data_Sheet_1.PDF]

Supplementary Material

Liver stiffness in Fontan patients: the effect of respiration and food intake

Annabell Braun†, Richard Mühlberg†, Marcus Fischer, Nikolaus A. Haas, Zora Meyer*

†These authors contributed equally to this work and share first authorship

*** Correspondence:** Zora Meyer: Zora.Meyer@med.uni-muenchen.de

1 Supplementary Data

	Group 1<br>Mean (SD)	Group 2<br>Mean (SD)	Relative Change (%)	P value
V. porta (cm/s)	19.85 (4.2)	19.89 (4.6)	0.2	0.975
V. hepatica (cm/s)	20.94 (5.9)	24.3 (6.8)	13.5	0.046 *
Tr. coeliacus (cm/s)	127.82 (23.4)	134.92 (34.3)	5.3	0.355
V. cava inferior (cm)	1,75 (0.3)	2.06 (0.4)	17.7	0.001 *

Supplementary Table 1. Flow velocities group 1 vs. group 2
